# Scalable MXene and PEDOT-CNT Nanocoatings for Fibre-Reinforced Composite De-Icing

**DOI:** 10.3390/ma15103535

**Published:** 2022-05-14

**Authors:** Gediminas Monastyreckis, Juan Tortosa Siles, Petr Knotek, Maria Omastova, Andrey Aniskevich, Daiva Zeleniakiene

**Affiliations:** 1 Department of Mechanical Engineering, Kaunas University of Technology, Studentu St. 56, 51424 Kaunas, Lithuania; j.tortosa@alumnos.upm.es (J.T.S.); daiva.zeleniakiene@ktu.lt (D.Z.); 2 Department of General and Inorganic Chemistry, University of Pardubice, Studentska 573, 532 10 Pardubice, Czech Republic; petr.knotek@upce.cz; 3 Polymer Institute, Slovak Academy of Sciences, Dubravska cesta 9, 845 41 Bratislava, Slovakia; maria.omastova@savba.sk; 4 Institute for Mechanics of Materials, University of Latvia, Jelgavas Str. 3, LV-1004 Riga, Latvia; andrey.aniskevich@pmi.lv

**Keywords:** nanocoatings, thermal imaging, de-icing, fibre-reinforced composites, MXenes, PEDOT-CNT

## Abstract

In this study, the de-icing performance is investigated between traditional carbon fibre-based coatings and novel MXene and poly(3,4-ethylenedioxythiophene)-coated single-walled carbon nanotube (PEDOT-CNT) nanocoatings, based on simple and scalable coating application. The thickness and morphology of the coatings are investigated using atomic force microscopy and scanning electron microscopy. Adhesion strength, as well as electrical properties, are evaluated on rough and glossy surfaces of the composite. The flexibility and electrical sensitivity of the coatings are studied under three-point bending. Additionally, the influence of ambient temperature on coating’s electrical resistance is investigated. Finally, thermal imaging and Joule heating are analysed with high-accuracy infrared cameras. Under the same power density, the increase in average temperature is 84% higher for MXenes and 117% for PEDOT-CNT, when compared with fibre-based coatings. Furthermore, both nanocoatings result in up to three times faster de-icing. These easily processable nanocoatings offer fast and efficient de-icing for large composite structures such as wind turbine blades without adding any significant weight.

## 1. Introduction

Wind turbine farms expand offshore and in cold regions due to higher wind speeds and air density, resulting in more energy production [1]. On the other hand, harsh climate conditions usually affect their functionality and lifetime [2,3]. Ice formation on the blades is one of the most severe problems, causing disturbed aerodynamic flow and reducing the wind turbine’s efficiency by up to 20% [4,5]. Additionally, it can cause excessive vibrations, unequal load distribution, and ice debris can damage the composite structures [6,7], leading to higher maintenance costs [8]. Ice preventing systems, so-called anti-icing, are based on various hydrophobic coatings, whereas de-icing is based on active heating using embedded heaters or hot air tubing [9,10]. Traditional de-icing systems are not always efficient and can result in a turbine’s power consumption of 5–10% [11]. Moreover, metal-based heaters usually experience face-sheet debonding and are vulnerable to lightning strikes [12,13], while carbon fibre (CF) based heaters require specific manufacturing integration during the moulding of the blade [14]. Until now, the demand for highly efficient, green, and scalable de-icing coating technology remains.

In the early 21st century, the fabrication of nanoparticles such as graphenes (GP) and carbon nanotubes (CNT) has led researchers to explore their new applicability [15,16,17]. Due to high electrical conductivity and low thickness, the formation of ultrathin coatings for Joule heating has gained attention [18]. Joule heating is a process when a current flowing through a resistor, in this case, a resistive nanocoating or carbon-based fabric, is transformed into heat, following Joule’s first law. Pure carbon-based nanocoatings possess hydrophobic properties and can be vulnerable to scratching. Therefore, conductive polymers such as poly(3,4-ethylenedioxythiophene) (PEDOT) can be used as shells of the nanoparticles, improving their electrical properties and stability [19]. The de-icing coating based on Joule heating using GP doped epoxy was analysed by Redondo et al. [20]. At the filler amount of 12 wt%, the heating rate of 13.6 °C/min was obtained under a power density of 0.125 W/cm^2^. Another study was based on GP coated glass fibre (GF) rowing, which increased by 80 °C after 180 s of 10 V heating [21]. Raji et al. [22] used epoxy-GP nanoribbon composite (5 wt%) and under 0.375 W/cm^2^ power density, achieved a heating rate of ~30 °C/min. Ten-layered aligned CNT coating of 6 µm thickness was also investigated for de-icing coatings by Yao et al. [23]. The coating showed 48 °C/min at 0.128 W/cm^2^. 

Another novel two-dimensional (2D) nanoparticle similar to GP is MXene. The most studied MXene particle Ti_3_C_2_T_z_ has shown excellent mechanical and electrical properties [24,25,26]. These properties can be influenced by different delamination methods [27]. Good compatibility and wettability properties between MXenes and epoxy-based composites were also reported [28,29,30]. In addition, MXenes are thermally stable and can heat up to 700 °C with minor degradation [31]. Easily processable methods of MXenes, and scalable application technology such as spray coating, are attractive for de-icing [32]. Despite this, Joule heating of MXenes was investigated only recently. Jia et al. [33] deposited Ti_3_C_2_ MXenes on a wood-pulp fabric grid followed by a hydrophobic methyltrimethoxysilane layer. By applying 0.174 W/cm^2^, a heating rate of 63 °C/min was achieved. Another experiment was performed by Li et al. [34], who prepared free-standing MXene-montmorillonite (MMT) thin film with the weight ratios of MXene to MMT of 90:10. The heating rate of the film was 100 °C/min under a power density of 4.58 W/cm^2^. The authors also reported the film to be stable under heating cycles.

Until now, only a few nanocoatings have been investigated for fibre-reinforced composite de-icing. In this work, for the first time, novel MXene and PEDOT-CNT nanocoatings are used. This research aimed to develop such nanocoatings using scalable water-based spray-coating methods and investigate their adhesion strength, flexibility, heating properties, and de-icing performance.

## 2. Materials and Methods

### 2.1. Materials and Specimens

Ti_3_C_2_T_z_ MXenes were prepared from Ti_3_AlC_2_ MAX phase with a particle size of <40 µm and purity of 98 wt% (MRC, Kyiv, Ukraine). The etching solvents were hydrochloric acid (37 wt%, Merck, Darmstadt, Germany) and lithium fluoride (>99 wt%, Merck, Darmstadt, Germany). MAX phase was stirred for 24 h at room temperature. The multilayer MXene sediment was further delaminated using 99 wt% LiCl (Merck, Darmstadt, Germany). The resulting solution was centrifuged 12 times at 3500 rpm and washed until the pH of the supernatant reached above 6. The concentration of the delaminated MXenes in the supernatant was reconcentrated from 0.34 mg/mL to 3 mg/mL, more suitable for spraying. PEDOT/(CNT+SO_3_H) aqueous paste with a 1:1 ratio (SYNPO, Pardubice, Czech Republic) was used for CNT coating preparation. The paste was reconcentrated to 0.33 mg/mL, which was more suitable for spraying without nozzle jamming.

Sandwich structured glass fibre-reinforced polymer (GFRP) composites were prepared by hand-layup and vacuum bagging methods. The samples consisted of 8 plies 163 g/m² twill-weave Interglas 92110 (Porcher Industries, Erbach, Germany), separated by 4 mm thick polyvinyl chloride foam (AIREX^®^ C70.75, Sins, Switzerland). The thermosetting epoxy resin Bisphenol F-epichlorohydrin and an amine curing agent were mixed at a ratio of 10:3 (Biresin^®^ CR-122-5, Sika, Zürich, Germany). All specimens were cured at room temperature for 24 h and post-cured in a convection oven for 4 h at 100 °C. Peel-ply fabric polyamide 6.6 (plain weave, 64 g/m²) and polyethylene film were used to make rough and glossy surfaces of the specimens, respectively. 

### 2.2. Coating Preparation

Unidirectional CF coating was made from 160 g/m^2^ plain weave fabric (R&G GmbH, Waldenbuch, Germany) by removing horizontal fibres and leaving four separated 3000 filament stripes for the wire connection (Figure S1a). The coating was used as the last layer of the composite and was curred using the vacuum bagging technique. Copper wires were glued to the coating by melting electrically conductive polylactic acid (PLA) (Protopasta, Protoplant, Inc., Vancouver, Canada), which was additionally covered with a thin layer of silver paint to reduce the contact resistance. The chopped CF coating (Figure S1b) was made from 3 mm CF strands (R&G GmbH, Germany) mixed with epoxy resin at a weight fraction of 1%. The coating was additionally covered with a 3000 CF “fork-type” filament grid and pressed and cured under the vacuum for the sedimentation of chopped fibres. The composite samples used for nanocoatings were firstly treated with Ar enriched plasma for 3 min at 80 W and 40 kHz, using a Zepto Diener low-pressure plasma cleaner (Diener electronic GmbH & Co. KG, Ebhausen, Germany). As a result, the water contact angle of the glossy composite surface was reduced from 70 to 25°, which determined more uniform nanocoatings. After the plasma treatment, water-based MXenes with a 3 mg/mL concentration were spray-coated several times, depending on the investigation (Figure S1c). Here, one-time sprayed means one coating layer made from 1 mL/85 cm^2^ spray yield, followed by a natural drying of 10 min. The PEDOT-CNT coating was made using the same technique as for MXene coating (Figure S1d). Both nanocoatings were prepared using a Sparmax HB-040 airbrush (Anest Iwata Sparmax Co., Ltd., Taiwan, China). After drying, the nanocoatings were applied with 7 mm silver paint stripes at the edges and additionally coated with a protective epoxy layer of 0.15 mm thickness. All four samples had the same heating area of 85 ± 1 cm^2^.

### 2.3. Characterisation and Testing

For the characterisation, 3-layer MXene and 5-layer PEDOT-CNT coatings were used. The morphology of the nanocoatings was characterised using scanning electron microscopy (SEM) (S-3400N, Hitachi, Tokyo, Japan). Silicon wafers (1 × 1 cm) were used as nanocoating substrates. The EDX measurements were performed using high vacuum mode, BRUKER Quantax EDS detector, and a working distance of 10 mm. The electron accelerating voltages were 5 keV for both magnifications. The thickness of MXene flakes and MXene coating was measured using atomic force microscopy (AFM) (Dimension Icon, Bruker, Billerica, Massachusetts, USA) based on the scratch method [35]. The topography of the surface was monitored in PeakForce quantitative nanoscale mechanical mode using ScanAsyst-Air tips (*k* = 0.4 N·m^−1^). The 10 × 10 µm sample images were recorded at a scanning frequency of 0.5 Hz. The roughness of the GFRP composite surface was analysed using 3D optical microscopy (Leica DVM6, Leica Microsystems, Wetzlar, Germany). 

Adhesion tests (Figure S2) of the coatings were performed on 8-ply GFRP specimens (15 × 3 cm) with glossy and rough surfaces using an Adheometer PM 420/63, under ISO-4624 standards. The square sheet electrical resistance (Ω/sq) dependencies on the nanocoating layers and substrate roughness were measured using a two-probe Fluke 287 True-RMS multimeter (Fluke Corporation, Everett, Washington, USA). Electrical resistance stability under ambient temperatures was performed in a temperature-controlled oven and a freezer. 

Three-point bending tests (Figure S3) were performed with sandwich structured GFRP samples (10 × 2.5 cm) using Tinius Olsen H25 KT universal testing machine (Tinius Olsen, Redhill, UK) under ISO-178 standards. Electrical resistances changes of tensiled and compressed nanocoating surfaces during the bending were measured between two silver paint stripes at the edges of the specimen (8.5 cm distance).

For thermal imaging analysis (Figure S4) and de-icing (Figure S5), the layers of nanocoatings were increased to 5 for MXenes and 8 for PEDOT-CNT, in order to achieve higher conductivity and perform heating in lower voltages. The investigation was performed with sandwich structured GFRP samples (10 × 10 cm) using an external power supply (Axiomet AX-12001 DBL, Transfer Multisort Elektronik, Łódź, Poland). The DC power (W) for all coatings was determined by first tested chopped CF coating, subjected to 5 V voltage (0.372 A current) and 10 V (0.744 A). Therefore, other coatings were also applied with the same power of 1.86 and 7.44 W. The heating time was chosen for 300 and 180 s, respectively, where the shorter time for 7.44 W was limited to the wire overheating. The temperatures were monitored using an infrared camera FLIR SC7500 (Teledyne FLIR LLC, Wilsonville, Oregon, USA), with a pixel pitch of 30 µm and ±1% temperature accuracy. For a de-icing experiment, 5 ± 1 mm thick ice (tap water) was naturally formed on top of the coating in a −15 °C freezer. The de-icing of the coatings was performed at room temperature under DC power of 7.44 W. 

## 3. Results

### 3.1. Coating Characterisation

**Figure 1:**
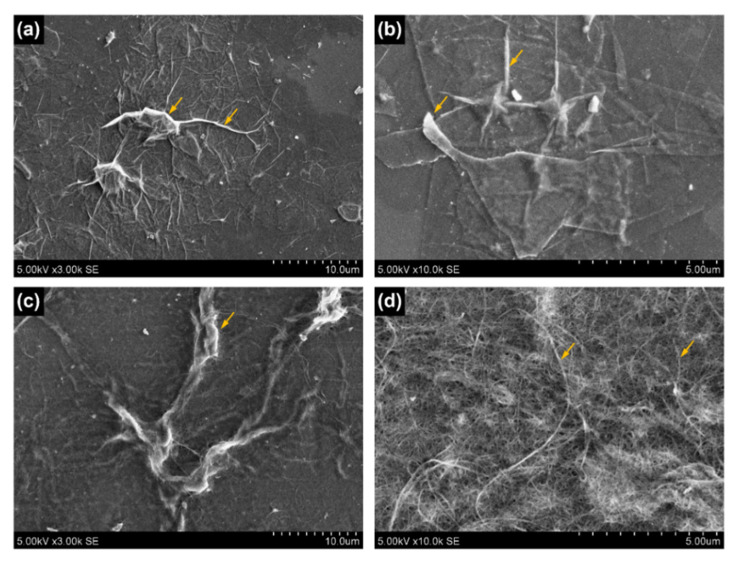
Scanning electron microscopy images of: (**a**) 3-layer MXene coating (**b**) magnified region; (**c**) 5-layer PEDOT-CNT coating (**d**) magnified region (yellow arrows clarified in the text).

The morphology of the nanocoatings, particle sizes, and structural integrity were studied using SEM. A three-layer MXene coating is presented in Figure 1a,b. Here, wrinkles were formed due to non-delaminated fragments and an overlapping structure (yellow arrows). The wrinkle extension of 10 µm from the fragment can be seen and is expected to have an effect on electrical conductivity and adhesion properties. Figure 1b shows a magnified region of MXene coating, where fully delaminated and overlapping flakes of 3–6 µm in size can be observed. We can also notice a non-adhered corner of the top flake, and sharp flake waviness, which suggests weak flexural rigidity of fully delaminated MXenes [36]. A five-layer PEDOT-CNT coating is presented in Figure 1c,d, where larger nanotube agglomerations and fibre-like bundles that stretch more than 30 µm are observed. Under higher magnification (Figure 1d), we can notice a more uniform single-walled CNT web-like structure. Although, the differences in CNT thickness and lengths are affected by conductive polymer PEDOT, which forms a shell around the tubes and acts as an adhesive matrix.

**Figure 2:**
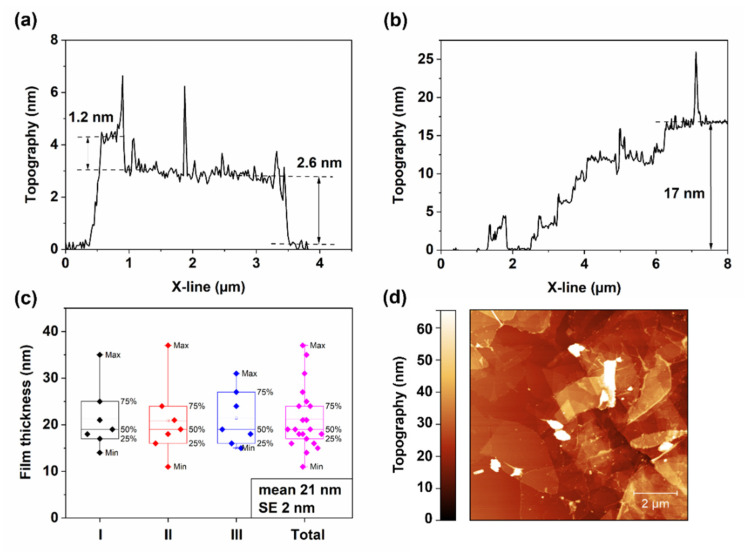
Atomic force microscopy analysis: (**a**) thickness measurement of delaminated single-layered and double-layered Ti_3_C_2_T_z_ MXene flakes; (**b**) 3-layer MXene coating thickness; (**c**) statistical thickness analysis of the coating; (**d**) topographical map of 3-layer MXene coating (close to scratch).

The thickness of Ti_3_C_2_ MXene flakes and three-layer coating was measured using AFM. In Figure 2a, we can notice a 0.5 µm size and 1.2 ± 0.1 nm thickness MXene flake on top of another flake. This represents a fully delaminated single-layered Ti_3_C_2_T_z_ flake, where T_z_ stands for functional surface groups (-O, -OH, and -F). Then, the bottom flake would roughly stand for a double-layered MXene flake with a thickness of 2.6 ± 0.1 nm, which is in good agreement with other studies [37]. The thickness of the MXene coating near the scratch could be evaluated in Figure 2b. Here, a gradual thickness increase of 2.3–2.7 nm can be observed at every 0.5–1 µm length, starting from the silicon wafer substrate and ending at 17 nm. Figure 2c demonstrates the statistical thickness scattering of a three-layer MXene coating, which was measured in three different sample places, with a mean value of 21 nm, standard error (SE) of 2 nm, and minimal and maximal values of 10 and 37 nm, respectively. In Figure 2d, a topographical map of the coating is presented. Here we can notice a smooth surface with several peaks above 60 nm, representing non-delaminated MXenes fragments. The AFM topography image shows that the coating is a well-defined overlapping structure with a lateral flake size of 2–6 µm, which corresponds with SEM analysis. PEDOT-CNT coating thickness was not measured using AFM due to its flexible and porous web-like structure, but it was roughly estimated for 150–250 nm, assuming a solution concentration of 0.33 mg/mL, one layer spray yield of 1 mL/85 cm^2^, the density of CNT, and the film’s porosity.

### 3.2. Electrical Properties and Adhesion Strength

**Figure 3:**
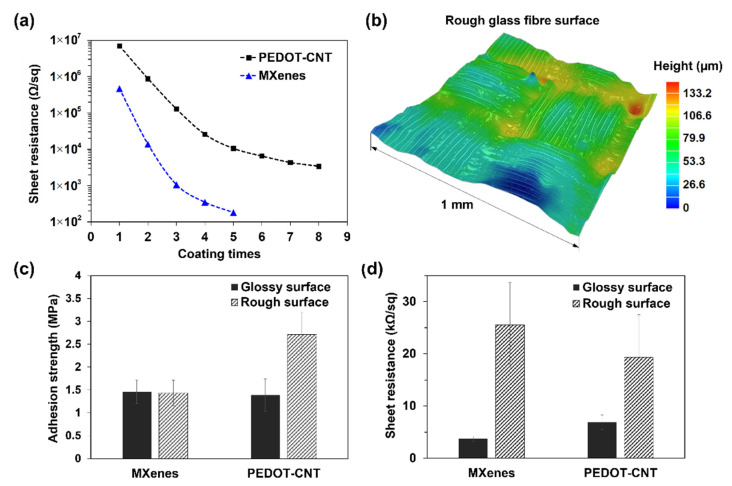
Electrical properties and adhesion strength of the nanocoatings: (**a**) electrical resistance dependence on the coating layers of MXenes and PEDOT-CNT; (**b**) 3D optical topography image of rough GFRP composite surface; (**c**) adhesion strength comparison between 3-layer MXene and 5-layer PEDOT-CNT on glossy and rough surfaces; (**d**) electrical resistance dependence on rough and glossy surfaces.

In order to develop a durable and compatible de-icing coating, one must approach its suitable electrical properties and adhesion strength. Firstly, the electrical properties of the coatings were investigated. The square sheet resistance dependence on the coating layers is shown in Figure 3a. Both coatings were prepared on plasma-treated GFRP samples with a glossy surface. MXene nanocoating showed that the resistance decreased roughly tenfold with every additional layer, starting from 500 kΩ/sq for the first layer and ending at around 200 ± 20 Ω/sq for the fifth layer. PEDOT-CNT coating did not show such a rapid decrease in resistance, and after the fourth layer, the slope slightly flattened. The first layer showed resistance of 7 MΩ/sq, and the fifth layer—10.5 ± 0.7 kΩ/sq, which is more than 50 times higher when compared with MXenes. It was not efficient to stack up more than eight layers of PEDOT-CNT due to insignificant changes in the resistance (Figure 3a), and the final values were 3 ± 0.3 kΩ/sq. Regarding the thickness of the coatings (see Section 3.1), the conductivity of the final MXene coating on the glossy surface was equal to ~1000 S/cm and PEDOT-CNT—15 S/cm.

It is known that the coating’s adhesion and electrical properties depend on the substrate’s wettability and roughness [38]. A 3D optical topography image (1 × 1 mm) of a rough GFRP surface is presented in Figure 3b, with a roughness of ~50 µm, while a glossy surface was ~1 µm. Adhesion strength results of three-layer MXene and five-layer PEDOT-CNT are presented in Figure 3c. The MXene coating showed roughly 1.5 MPa for both glossy and rough surfaces, while the PEDOT-CNT coating showed 1.4 and 2.7 MPa, respectively. The PEDOT-CNT fracture behaviour on glossy and rough surfaces suggests that the adhesive glue went through the CNT web-like structure and adhered directly with the GFRP sample, resulting in almost twice higher strength. This observation is also supported by the high increase in electrical resistance during the metal holder glueing. In contrast, the adhesion strength of MXene coating on both surfaces was similar, and we could expect Mxene–MXene interaction to be the main factor, as was previously reported [39]. In comparison, non-coated GFRP composite resulted in up to three times higher adhesion strength than nanocoatings due to a strong epoxy–epoxy interaction.

The electrical resistance dependence on substrate roughness is shown in Figure 3d. Here, electrical resistance differs up to seven times for MXenes, and only three times for CNTs when comparing glossy and rough surfaces. These results suggest that CNTs can easily deform and shape against the substrate without losing conductivity, while 2D MXene flakes are less flexible.

### 3.3. Ambient Temperature and Flexibility

**Figure 4:**
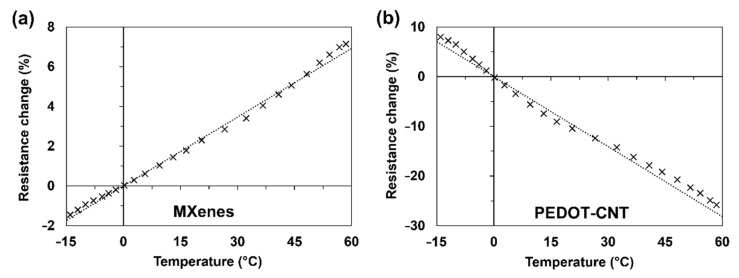
Electrical resistance changes under the ambient temperature of: (**a**) MXene and (**b**) PEDOT-CNT coatings.

It is important to understand how the electrical properties of the coatings behave in different ambient temperatures and under mechanical deformations during the exploitation. In Figure 4, the electrical resistance changes in the −15–60 °C temperature conditions are presented. In Figure 4a, MXene nanocoating shows a stable resistance increase at the ratio of 1.2% per 10 °C. In contrast, PEDOT-CNT coating shows a decrease of 4.7% per 10 °C (Figure 4b). Such behaviour of MXenes is similar to metals, while the opposite behaviour of PEDOT-CNT is similar to carbon-based materials.

**Figure 5:**
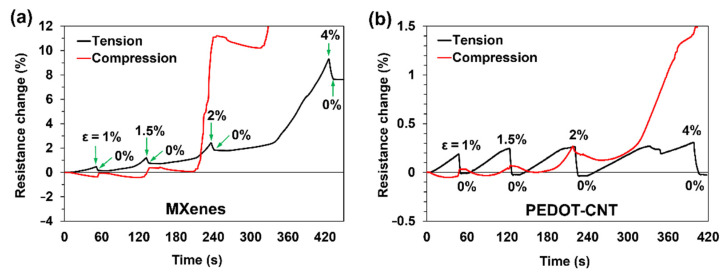
Resistance changes of tensiled and compressed surfaces under three-point bending at different flexural strains of: (**a**) MXene and (**b**) PEDOT-CNT coatings.

Another important characteristic of de-icing heaters is the electrical resistance stability during deformation. The resistance was monitored under three-point bending separately for lower and upper surfaces, deforming under tension and compression, respectively (Figure 5). In addition, the bending test included four cycles, each reaching ever higher deflection values of 2, 3, 4, and 8 mm, resulting in flexural strain (*ε*) of 1, 1.5, 2, and 4%, respectively. Tensiled MXenes showed a slight increase in resistance, while the values were opposite under compression (Figure 5a). When the deflection was restored to 0 mm after each cycle, the coating’s resistance did not return to the initial value, as was previously reported [39]. In contrast, PEDOT-CNT coating returned almost exactly to the initial resistance value after each cycle (Figure 5b). At ~3% compression strain, both coatings showed a steep electrical resistance increase due to the initial cracking of the composite’s top laminate, which together damaged the coating. However, MXene coating resulted in a sudden resistance increase of 30% (not shown in Figure 4a), while PEDOT-CNT coating resulted in only a 1.5% increase. Such results indicate that MXenes are adhered to the substrate and will likely crack together with the surface fibres. The piezo-resistive mechanism of MXenes is based on flake-to-flake conductivity loss. In contrast, PEDOT-CNT coating is more flexible, and a web-like nanotube structure is expected to realign or deform, and in such a way, the tube-to-tube conductivity is maintained. Despite the resistance change due to ambient temperature or deformations, to perform the de-icing procedure under the same power density, one can increase the applied voltage following Joule’s first law.

### 3.4. Thermal Imaging and De-Icing

**Figure 6:**
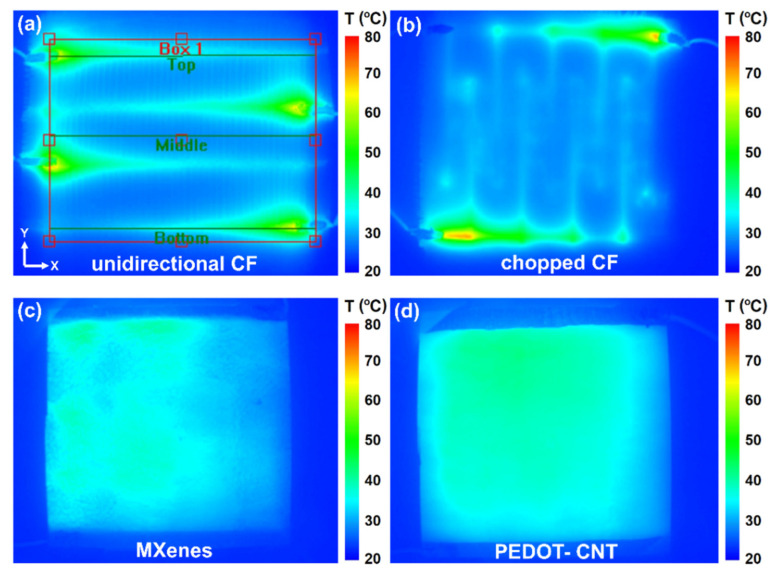
Thermal imaging analysis under 1.86 W power and 300 s of: (**a**) unidirectional CF; (**b**) chopped CF; (**c**) 5-layer MXene and (**d**) 8-layer PEDOT-CNT coatings.

Thermal imaging was performed to check coatings integrity, temperature leaks in high resistive areas, and de-icing efficiency. The heating performance of the coatings was compared using the same power density (W/cm^2^). In Figure 6, thermal images after continuous heating at room temperature for 300 s with 1.86 W are presented. In the unidirectional CF sample, temperature concentrated near wire connections (Figure 6a), which was caused by high resistivity differences between conductive PLA (0.15 Ωm), silver paint (10^−7^ Ωm), and CF (10^−5^ Ωm). Chopped CF coating showed similar behaviour (Figure 6b), where non-uniformly heated areas appeared due to unevenly distributed CF strands, with temperatures of up to 70 °C. In contrast, MXene nanocoating (Figure 6c) did not possess wire overheating due to the higher resistance of MXenes (245.9 Ω). Additionally, the temperature accumulated in the middle of the sample, although the right side was slightly colder due to hand-spraying defects. PEDOT-CNT coating shown in Figure 6d resulted in the best temperature distribution, possibly caused by a web-like structure and more coating layers. In addition, due to the high coating resistance of PEDOT-CNT (3094 Ω), no wiring overheating was observed.

**Figure 7:**
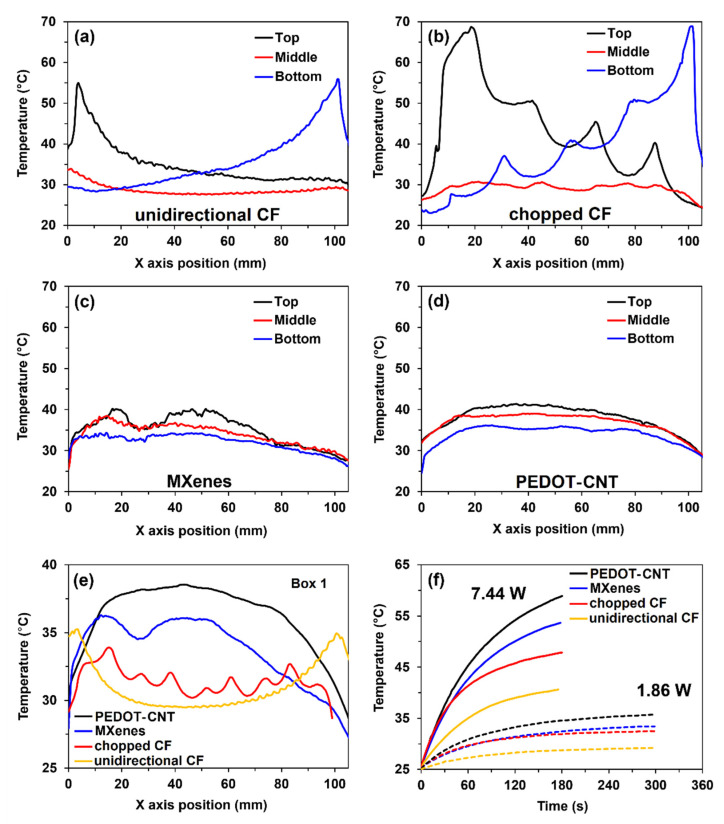
Comparison of temperatures along three linear sections under 1.86 W and 300 s of: (**a**) unidirectional CF; (**b**) chopped CF; (**c**) MXenes; (**d**) PEDOT-CNT; (**e**) average temperature results of the coatings across the sample length under 1.86 W and 300 s; (**f**) average temperature increase overtime under 1.86 and 7.44 W.

Joule’s heating (1.86 W, 300 s) is also studied along three linear sections named “Top, Middle, Bottom”, and in the area (85 cm^2^) named “Box 1”, shown in Figure 6a. Unidirectional CF coating (Figure 7a) shows temperature concentrations at the edges of up to 55 °C, while temperatures in the middle of the sample are relatively uniform and reach 29 °C. A sample with chopped CF shown in Figure 7b follows a similar trend. Here, the temperature at the wiring reached 68 °C and in the middle 31 °C. We can also notice temperature peaks at the CF filament grid connections due to higher contact resistance. In contrast, MXene and PEDOT-CNT coatings (Figure 7c,d) result in a more even temperature distribution along the linear sections, scattering between 34 and 41 °C. However, PEDOT-CNT coating showed smoother translation between individual data points, indicating better heat distribution in web-like CNT structure than overlapping 2D MXene flake structure.

The average areal temperatures (Box 1) of all four coatings after 300 s under 1.86 W, are compared in Figure 7e. The heat in unidirectional CF coating mostly accumulates at the sample sides, while the chopped CF sample shows a slightly improved heat distribution but with more chaotic temperature jumps. In comparison, both MXene and PEDOT-CNT nanocoatings produce more heat, with CNTs being slightly better. The average coating temperature (Box 1) increases over time is presented in Figure 7f. Here, samples are additionally tested at four times higher power load (7.44 W) but shorter time—180 s, to avoid wire overheating of CF coatings. The temperature results of unidirectional CF, chopped CF, MXenes, and PEDOT-CNT coatings are 40.6, 47.9, 53.7, and 58.9 °C, respectively. In addition, the initial heating rate of the coatings is equal to 10.1, 16.3, 17.4, and 20.39 °C/min, respectively. Such results suggest the difference in the coating’s efficiency, and when comparing maximum temperatures achieved over the same time, MXenes outperform traditional CF coatings by 84%, and PEDOT-CNT outperforms by 117%. The reason for such results is the much smaller thickness of the nanocoatings and fewer heat losses in the wires.

De-icing time of the coatings was evaluated under a 7.44 W power load. The de-icing time, shown in Table 1, was recorded at the moment when ice detached from the vertically positioned sample (Figure S5). A unidirectional CF coating was de-iced after 17 min when most of the ice had melted. Such prolonged time was caused by several low-heated areas, which kept the ice adhered. A similar issue appeared for chopped CF coating due to uneven heat distribution, and it took 13 min for the ice to detach. For both MXene and PEDOT-CNT coatings, the de-icing time was similar. At roughly ~5 min, the whole ice detached as one piece. These results show that nanocoatings can offer faster de-icing and lower energy consumption when compared to traditional CF-based heaters. With simple spray-coating techniques, the nanocoatings could be easily integrated into the manufacturing process or applied to existing composite structures already under exploitation. Due to their ultrathin thickness of a few dozen nanometers, MXene or PEDOT-CNT nanocoatings could also be considered for high-efficiency thermal heaters, e.g., in space applications.

## 4. Conclusions

The characterisation of thickness and morphology was performed using AFM and SEM, respectively. The results showed that using fully delaminated MXenes, a uniform and highly conductive (1000 S/cm) nanocoating of up to 37 nm thickness can be obtained.

The coating’s adhesion strength and electrical properties were investigated on rough and glossy surfaces of the composite. For MXenes, the resistance was up to seven times higher on a rough surface than on a glossy, and only three times higher for PEDOT-CNT. The adhesion strength between MXene and PEDOT-CNT was marginal.

The influence of ambient temperature on the coating’s electrical properties was analysed in the −15 and 60 °C range. MXene coating showed a stable electrical resistance increase at the ratio of 1.2% per 10 °C, while PEDOT-CNT resulted in the opposite and higher ratio—a decrease of 4.7% per 10 °C.

Under three-point bending, MXene coating’s electrical response was more sensitive to deformation and resulted in a permanent resistance increase, while PEDOT-CNT coating was more electrically stable and flexible.

Both MXene and PEDOT-CNT coatings resulted in uniform heat distribution throughout the sample and showed no wire-overheating, which was observed in traditional carbon fibre coatings. Additionally, the average coating temperature increase under the same power density and time was 84% higher for MXenes, and 117% for PEDOT-CNT. Therefore, both nanocoatings resulted in up to three times faster de-icing when compared to fibre-based coatings.

These results demonstrate that MXenes and PEDOT-CNT can be used as viable materials for scalable and easily processable de-icing nanocoatings for fibre-reinforced composites. Furthermore, the nanocoatings’ electrical properties and heating performance justify further investigation of onsite testing with full-scale heaters.

## Figures and Tables

**Table 1:** De-icing time of different coatings at the same power density of 0.088 W/cm (7.44 W).

Coating Type	Resistance [Ω]	Heating Rate [℃/min]	De-Icing Time [min]
unidirectional CF	2.1	10.1	17 ± 1
chopped CF	13.4	16.3	13 ± 1
5-layer MXenes	245.9	17.7	5 ± 0.5
8-layer PEDOT-CNT	3093.9	20.3	5 ± 0.5

## Data Availability

Not applicable.

## Supplementary Materials

The following supporting information can be downloaded at: https://www.mdpi.com/article/10.3390/ma15103535/s1, Figure S1: Sandwich structured GFRP composite samples coated with: (a) unidirectional CF; (b) chopped CF; (c) Ti_3_C_2_T_z_ MXenes; (d) PEDOT-CNT; Figure S2: Adhesion test setup; Figure S3: Three-point bending test setup; Figure S4: Thermal imaging test setup; Figure S5: De-icing test: (a) ice formation in a −15 °C freezer (horizontal). De-icing (vertically positioned sample) at room temperature under 7.44 W power of: (b) unidirectional CF coating after 12 min of heating; (c) chopped CF coating after 7 min; (d) MXene coating after 1 min; (e) a fully de-iced MXene coating after 5 min; (f) detached ice after 5 min of MXene coating.
