# Supplementary material for: Scalable MXene and PEDOT-CNT Nanocoatings for Fibre-Reinforced Composite De-Icing
Source: Materials (Basel). 2022 May 14;15(10):3535. doi: 10.3390/ma15103535 (PMC9144452; doi:10.3390/ma15103535)
Supplement: Supplementary file 1 [file materials-15-03535-s001.zip › materials-1706478-supplementary.pdf]

Supplementary Materials

# Scalable MXene and PEDOT-CNT Nanocoatings for Fibre-Reinforced Composite De-Icing


**Gediminas Monastyreckis [1,*], Juan Tortosa Siles [1], Petr Knotek [2], Maria Omastova [3], Andrey Aniskevich [4] and Daiva Zeleniakiene [1]**

[1] Department of Mechanical Engineering, Kaunas University of Technology, Studentu St. 56, 51424 Kaunas, Lithuania; j.tortosa@alumnos.upm.es (J.T.S.); daiva.zeleniakiene@ktu.lt (D.Z.)
[2] Department of General and Inorganic Chemistry, University of Pardubice, Studentska 573, 532 10 Pardubice, Czech Republic; petr.knotek@upce.cz
[3] Polymer Institute, Slovak Academy of Sciences, Dubravska cesta 9, 845 41 Bratislava Slovakia; maria.omastova@savba.sk
[4] Institute for Mechanics of Materials, University of Latvia, Jelgavas Str. 3, LV-1004 Riga, Latvia; andrey.aniskevich@pmi.lv
* Correspondence: gediminas.monastyreckis@ktu.edu; Tel.: +370-61104007


*2.2. Coating preparation*

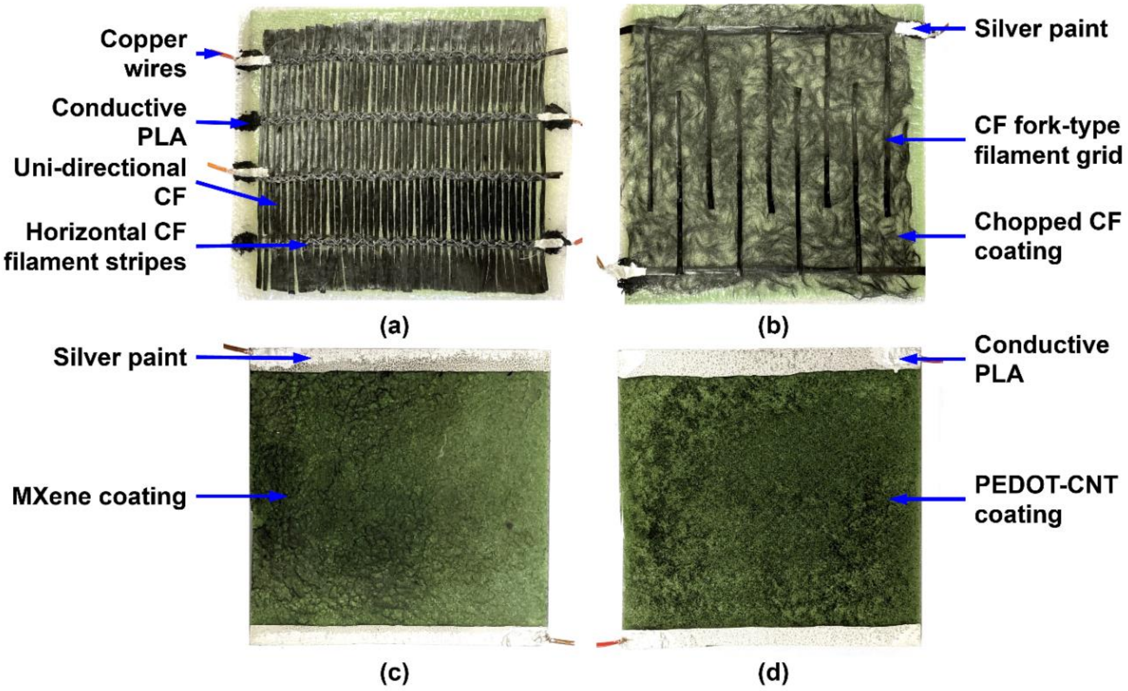


**Figure S1.** Sandwich structured GFRP composite samples coated with: (**a**) unidirectional CF; (**b**) chopped CF; (**c**) $Ti_3C_2T_z$ MXenes; (**d**) PEDOT-CNT.

*2.3. Characterisation and testing*

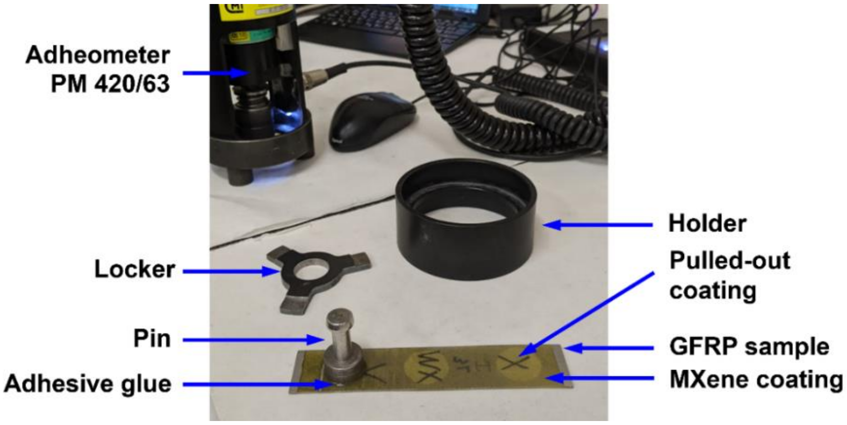


**Figure S2.** Adhesion test setup.

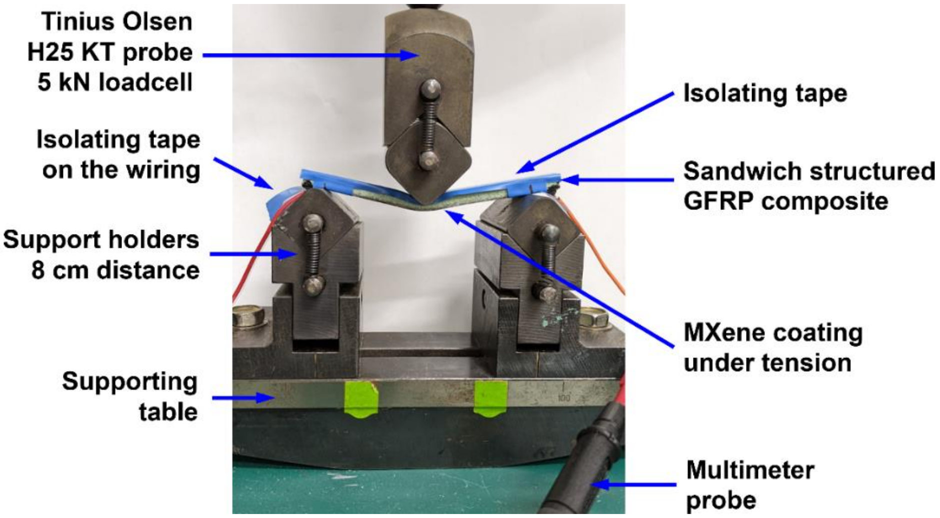


**Figure S3.** Three-point bending test setup.

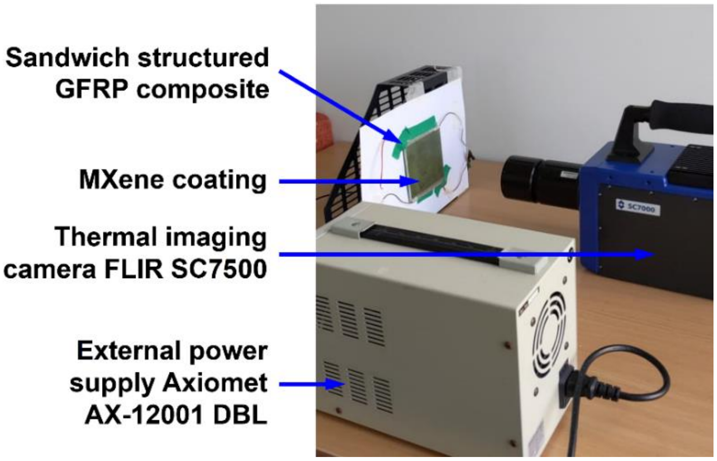


**Figure S4.** Thermal imaging test setup.

*3.4. Thermal imaging and de-icing*

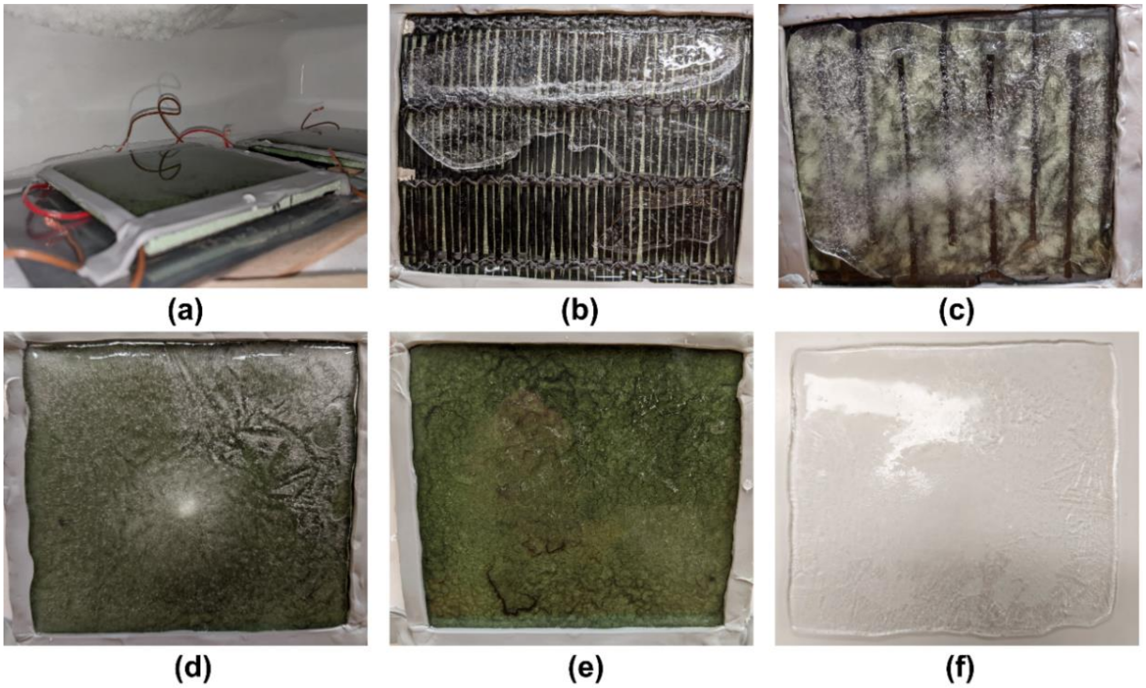


**Figure S5.** De-icing test: (**a**) ice formation in a -15 °C freezer (horizontal). De-icing (vertically positioned sample) at room temperature under 7.44 W power of: (**b**) unidirectional CF coating after 12 min of heating; (**c**) chopped CF coating after 7 min; (**d**) MXene coating after 1 min; (**e**) a fully de-iced MXene coating after 5 min; (**f**) detached ice after 5 min of MXene coating.
